# Predictive power of gait and gait-related cognitive measures in amnestic mild cognitive impairment: a machine learning analysis

**DOI:** 10.3389/fnhum.2023.1328713

**Published:** 2024-01-29

**Authors:** Cosimo Tuena, Chiara Pupillo, Chiara Stramba-Badiale, Marco Stramba-Badiale, Giuseppe Riva

**Affiliations:** ^1^Applied Technology for Neuro-Psychology Lab, IRCCS Istituto Auxologico Italiano, Milan, Italy; ^2^Department of Geriatrics and Cardiovascular Medicine, IRCCS Istituto Auxologico Italiano, Milan, Italy; ^3^Humane Technology Lab, Università Cattolica del Sacro Cuore, Milan, Italy

**Keywords:** amnestic mild cognitive impairment, gait abnormalities, cognitive dysfunction, Alzheimer’s disease, artificial intelligence, motor system

## Abstract

**Introduction:**

Gait disorders and gait-related cognitive tests were recently linked to future Alzheimer’s Disease (AD) dementia diagnosis in amnestic Mild Cognitive Impairment (aMCI). This study aimed to evaluate the predictive power of gait disorders and gait-related neuropsychological performances for future AD diagnosis in aMCI through machine learning (ML).

**Methods:**

A sample of 253 aMCI (stable, converter) individuals were included. We explored the predictive accuracy of four predictors (gait profile plus MMSE, DSST, and TMT-B) previously identified as critical for the conversion from aMCI to AD within a 36-month follow-up. Supervised ML algorithms (Support Vector Machine [SVM], Logistic Regression, and k-Nearest Neighbors) were trained on 70% of the dataset, and feature importance was evaluated for the best algorithm.

**Results:**

The SVM algorithm achieved the best performance. The optimized training set performance achieved an accuracy of 0.67 (sensitivity = 0.72; specificity = 0.60), improving to 0.70 on the test set (sensitivity = 0.79; specificity = 0.52). Feature importance revealed MMSE as the most important predictor in both training and testing, while gait type was important in the testing phase.

**Discussion:**

We created a predictive ML model that is capable of identifying aMCI at high risk of AD dementia within 36 months. Our ML model could be used to quickly identify individuals at higher risk of AD, facilitating secondary prevention (e.g., cognitive and/or physical training), and serving as screening for more expansive and invasive tests. Lastly, our results point toward theoretically and practically sound evidence of mind and body interaction in AD.

## 1 Introduction

Gait and balance are no longer considered solely as physical activity, but rather as multifaceted complex processes involving the integration of motor, perceptual, and cognitive functions (Montero-Odasso et al., 2012; Ahn et al., 2023). More specifically, executive functions, which encompass attentional control, cognitive flexibility, psychomotor processing, inhibition, and goal setting, share a comprehensive neural network with motor abilities and gait control (Leisman et al., 2016). This network includes the prefrontal cortex, medial temporal lobe, and nigrostriatal system. Additionally, it encompasses anatomical structures like ventricles, cerebellum, white matter tracts, and the parietal lobes (Scherder et al., 2007; Wennberg et al., 2017; Crook et al., 2020).

In the past, healthcare professionals and researchers used to conduct gait assessments and cognitive assessments as distinct evaluations for older adults (Montero-Odasso et al., 2012). However, recent findings from clinical practice, epidemiological research, and clinical trials have provided growing evidence that gait and cognition are interconnected in the elderly (Woollacott and Shumway-Cook, 2002). Indeed, a substantial body of research consistently indicates a connection between gait abnormalities and early signs of cognitive decline, even among cognitively healthy individuals (Mielke et al., 2013; Savica et al., 2017). This relationship underscores the intricate interplay between physical and cognitive health. Gait disorders include slow, unstable, shifting, staggering, and/or asymmetrical walking as a result of neurological, musculoskeletal, and/or other acquired medical conditions (Verghese et al., 2008; Pirker and Katzenschlager, 2017). For instance, it was found that Mild Parkinsonian Signs (MPS) were a strong indicator for the development of dementia in the future. In both unaltered and statistically adjusted examinations, individuals exhibiting MPS were twice as prone to experiencing the onset of dementia when compared to their counterparts who did not exhibit these neurological indications (Louis et al., 2010). Changes in gait can guarantee potential early indicators of underlying cognitive issues, providing valuable insights into an individual’s neurological well-being before more apparent cognitive decline symptoms manifest (Ahn et al., 2023). Gait disorders can be assessed through visual inspection or quantitatively (e.g., speed, stride length, swing, and stance time), depending on how they affect the observed gait abnormalities. While the latter requires technological equipment that can be used to further differentiate individuals based on their cognitive status, the former is a helpful and reliable tool in routine therapeutic practice (Verghese et al., 2008).

For example, there is substantial evidence indicating that gait irregularities have the potential to forecast a gradual deterioration in cognitive function as assessed by the Digit Symbol Substitution Test (DSST), which evaluates executive functions, psychomotor speed, and attention (Kaufman, 1983), as demonstrated in numerous studies (Rosano et al., 2005; Inzitari et al., 2007; Mielke et al., 2013). Moreover, gait abnormalities are predictive of a reduction in both divided attention and cognitive flexibility, as assessed by the Trail Making Test part B (TMT-B) (Mielke et al., 2013). Furthermore, the presence of gait abnormalities is longitudinally linked to a decline in global cognitive assessments (Alfaro-Acha et al., 2007; Taniguchi et al., 2012). Gait disorders have been identified as one of the contributing factors to the development of dementia (Verghese et al., 2008).

In recent years, there has been a growing focus on the milder end of the cognitive staging, which encompasses the spectrum from normal aging to Alzheimer’s Disease (AD). It is increasingly recognized that there exists a transitional phase between normal aging and the clinical diagnosis of very early-stage AD. This intermediate stage has been referred to by various terms, including Mild Cognitive Impairment (MCI) (Petersen, 2004, 2011). In recent years, there has been a growing focus on amnestic MCI (aMCI), which refers to individuals experiencing memory loss beyond what is typical for their age and education. These individuals are also at a higher risk of developing AD compared to those with non-amnestic MCI (Petersen, 2011). Increased gait variability was recently linked to dementia, especially AD dementia and PD dementia, according to multisite research of 500 older persons with various neurodegenerative diseases. Although rhythm and postural control domains were similarly linked to dementias and MCI, only gait variability could reliably identify and categorize people with AD (Pieruccini-Faria et al., 2021).

Moreover, in a study (utilizing accelerometers to analyze gait patterns in individuals with normal cognition (NC), MCI, and AD, it was observed that the AD group exhibited significantly lower velocity and step length compared to both MCI and NC subjects. Notably, the inclusion of dual-task testing, where subjects were instructed to count backward from 100 to 0 while undergoing gait assessment, proved to be crucial in distinguishing between MCI and NC individuals (Muurling et al., 2020). These findings underscore the early onset of gait dysfunction in the AD spectrum and underscore the value of gait assessments as a potential biomarker for predicting transitions.

A recent longitudinal retrospective study underlined that the presence or the severity of gait and/or balance disturbances was associated with an increased risk of AD in a sample of aMCI. Therefore, community-dwelling older adults with aMCI may need frequent evaluation by nurses to detect possible risk factors for cognitive decline, particularly in cases involving gait and/or balance issues (Ahn et al., 2023). Early evidence by Tuena et al. (2023) highlighted that gait abnormalities identified through routine neurological gait examinations are linked to distinct trends in cognitive test performance over time in individuals with aMCI. Specifically, when compared to the group with normal gait, those with abnormal gait experienced a more rapid decline in attention (DSST) and overall cognitive function (Mini-Mental State Examination [MMSE]) tests. Significantly, TMT parts A (TMT-A) and TMT-B exhibited unique declines over time in the abnormal gait group, not observed in the normal gait group. Crucially, the presence of gait disorders [hazard ratio (HR) = 1.70] and declines in the performance of three gait-related cognitive tests (MMSE, HR = 1.09; DSST, HR = 1.03; TMT-B, HR = 1.01) were significantly associated with a higher risk of developing AD dementia in the overall aMCI Alzheimer’s Disease Neuroimaging Initiative (ADNI) population.

In recent years, machine learning (ML) models have been developed to make prognostic/diagnostic predictions to improve AD clinical practice (Battista et al., 2020). Machine learning models can be used to improve our understanding of the conversion from MCI to AD dementia by predicting clinical progressions and identifying individuals at risk. These models utilize different features (clinical, biological, and neuroimaging data, biomarker positivity, neuropsychological tests, and comorbidity information) to estimate the probabilities of progression (Pang et al., 2023). By incorporating longitudinal information encoded in efficient markers, machine learning frameworks can differentiate between progressive and non-progressive MCI subjects, aiding in developing personalized strategies for preventing or slowing the progression of dementia (Battista et al., 2020; Bucholc et al., 2023). By analyzing large datasets and applying machine learning algorithms, these models can identify patterns and relationships that may not be apparent through traditional statistical methods. Interpretable machine learning algorithms can also be used to develop predictive algorithms for individual conversion to dementia, considering complex patterns and interactions between variables (Chun et al., 2022). These machine learning approaches can contribute to early intervention and the selection of appropriate treatments for individuals at high risk of developing dementia (Dhakal et al., 2023).

Based on these premises, we further explored the predictive value of the gait profile and gait-associated neuropsychological measures that have been identified in previous work (Tuena et al., 2023). The aim of this study was to evaluate the potential of a neuropsychological assessment combined with a gait objective exam in predicting the transition from aMCI to AD dementia through machine learning algorithms. In the present study, we developed an ML model to predict conversion from aMCI to AD using relevant features (MMSE, DSST, TMT-B, and gait profile). We investigated the role of these three neuropsychological tests and gait profiles in the automatic classification of aMCI subjects who converted or did not convert to AD within 36 months. We developed an ML model to assess the accuracy of predicting conversion by using our features.

## 2 Materials and methods

### 2.1 Study sample

Data used in the preparation of this article were obtained from the Alzheimer’s Disease Neuroimaging Initiative (ADNI) database^1^. The ADNI was launched in 2003 as a public-private partnership, led by the principal investigator, Michael W. Weiner, MD. The primary goal of ADNI has been to test whether serial magnetic resonance imaging (MRI), positron emission tomography (PET), other biological markers, and clinical and neuropsychological assessment can be combined to measure the progression of MCI and early AD^2^.

**FIGURE 1:**
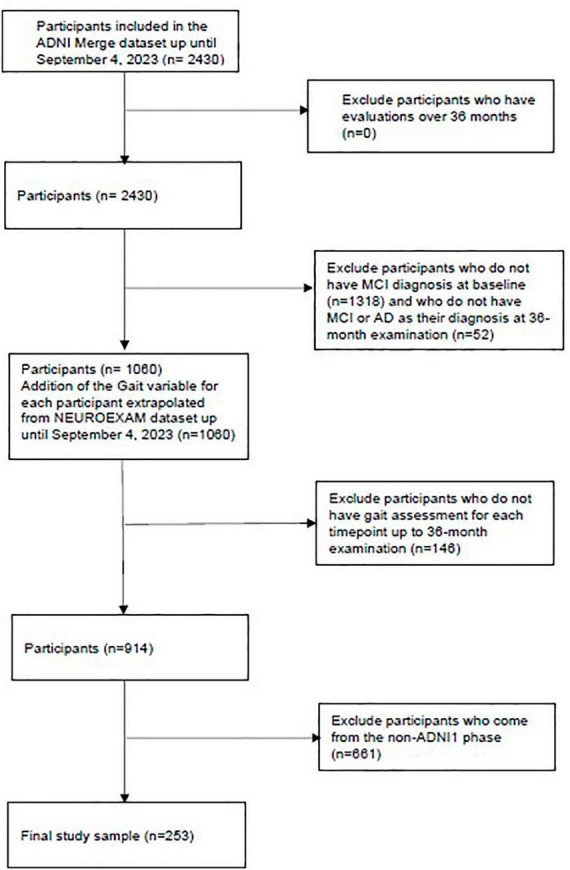
Flowchart of patient inclusion.

We extrapolated 253 participants from the ADNI phase 1 (ADNI1) database (recruited in North America)^3^: 107 (42.29%) aMCI individuals did not convert to dementia from baseline to the last time point considered in this study (36 months, aMCI stable [aMCIs]) and 146 (57.71%) converted to dementia (aMCI converters [aMCIc]). The data used for this paper are a subset of the full data set from the ADNIMERGE package (n = 2430): initially, all subjects diagnosed with MCI at baseline and converting to AD within 36 months (at months 6, 12, 18, 24, 36) were extrapolated (n = 1060). This allowed the variable of final diagnosis (within 36 months) to be created. We excluded any MCI patients who dropped out during the follow-up and included patients who received the AD diagnosis during the five-time points or remained stable until month 36. Thus, the data were divided into two groups, indicating the final diagnoses. Those who received a diagnosis of AD within 36 months were placed in class 1 (aMCIc), while the others were placed in class 0 (aMCIs). The detailed steps of the extrapolated sample selection are shown in Figure 1. Criteria for ADNI eligibility and diagnostic classifications are described at https://adni.loni.usc.edu/wp-content/themes/freshnews-dev-v2/documents/clinical/ADNI-1_Protocol.pdf.

The following variables were extrapolated: baseline demographics (age, sex, education level), neuropsychological test scores at baseline (MMSE, DSST, and TMT-B), and gait profile at baseline (classified as normal/abnormal). The prevalence of gait abnormalities in the whole sample (N = 253) was 9.9%. Table 1 shows the sociodemographic and relevant clinical variables taken into consideration in this study.

**TABLE 1:** Summary of socio-demographic and relevant clinical variables at baseline of the aMCI group by final diagnosis (within 36 months).

	aMCIs = 107				aMCIc = 146		
	**N**	**Mean**	**SD**	**N**	**Mean**	**SD**	***p*-value**
Age (years)	107	75.22	7.01	146	74.83	7.15	0.666
Gender							0.333
Female	36	33.64%		59	40.41%		
Male	71	66.36%		87	59.59%		
Education (years)	107	15.67	2.96	146	15.73	2.82	0.884
Gait type							1.000
Normal	96	89.72%		132	90.41%		
Abnormal	11	10.28%		14	9.59%		
MMSE (points)	107	27.68	1.70	146	26.66	1.72	< 0.001
DSST (points)	107	40.74	10.91	146	34.92	10.53	< 0.001
TMT-B (seconds)	107	113.97	63.76	146	146.51	79.28	< 0.001

aMCI, Amnestic Mild Cognitive Impairment; aMCIs, aMCI stable; aMCIc, aMCI converters; MMSE, Mini-Mental State Examination; DSST, Digit Symbol Substitution Test; TMT-B, Trail Making Test part B. Mean and SD are reported. For numerical variables, a T-test is performed, while for categorical variables, a Chi-squared test is used. Bold values represent significant *p*-values.

In the ADNI protocol (Mueller et al., 2005), individuals with MCI were diagnosed according to the MCI Petersen criteria (2011). The inclusion criteria for the aMCI ADNI group were as follows: a memory complaint by subject or caregiver that is verified by a study partner; abnormal memory function documented by a score below the cut off (adjusted for instruction) on the logical memory II sub-scale from the Wechsler memory scale−revised; MMSE score in the range 24–30; Clinical Dementia Rating (CDR) score of 0.5; general cognition and functional performance were sufficiently preserved such that a diagnosis of AD was not made at the time of the screening visit; the modified Hachinski score ≤ 4; age in the range 55–90 years old; stable assumption of permitted medications at least 4 weeks prior to screening; Geriatric Depression Scale (GDS) score < 6; the presence of adequate visual and auditory acuity to allow neuropsychological testing and good general health with no additional diseases; availability and ability to complete all baseline assessments and participate in a 3-year protocol; availability to undergo MRI 1.5 Tesla neuroimaging and provide DeoxyriboNucleic Acid (DNA) for Apolipoprotein E (ApoE) assessments and banking as well as plasma samples at protocol specified time points; completed 6 grades of education (or had a good work history sufficient to exclude intellectual disability); fluent in English or Spanish.

The exclusion criteria for the aMCI ADNI group were as follows: any significant neurologic disease other than suspected incipient AD or history of significant head trauma followed by persistent neurologic defaults or known structural brain abnormalities; evidence of infection, infarction, or other focal lesions, multiple lacunes or lacunes in a critical memory region; presence of pacemakers, aneurysm clips, artificial heart valves, ear implants, metal fragments or foreign objects in the eyes, skin or body; recent diagnosis (within the past year) of major depression or bipolar disorder; manifestations (within the last three months) of psychotic features, agitation or behavioral problems; history of schizophrenia; history of alcohol or substance abuse or dependence (within the past 2 years); any significant systemic illness or unstable medical condition; clinically significant abnormalities in vitamin B12, rapid plasma regain test, or thyroid function tests; residence in skilled nursing facility; current use of specific psychoactive medications and warfarin; participation in clinical studies involving neuropsychological measures being collected more than one time per year.

This information was extracted from the ADNI1 (Mueller et al., 2005) clinical protocol section^4^. Ethical approval for data collection and sharing was given by the institutional review boards of the participating institutions in the ADNI.

### 2.2 Gait screening and neuropsychological measures

At screening visits, accredited specialists performed a neurological gait evaluation in accordance with the ADNI clinical protocol to make sure patients were eligible before the baseline assessment. After a visual evaluation of gait patterns (e.g., walking for a short distance) and balance (i.e., tandem walk, Romberg test), the ADNI specialist classified the gait as normal or abnormal. In our study, the gait variables mentioned above were extrapolated from the neurological examinations (NEUROEXM.csv) referring to ADNI1 protocols.

The baseline assessment included a range of clinical and medical information and the administration of a series of neuropsychological tests (e.g., MMSE, DSST, and TMT). We extrapolated neuropsychological test scores (MMSE, DSST, and TMT-B) for the ADNI1 protocol. This information was retrieved from the ADNI file regarding the ADNIMERGE package (ADNIMERGE.csv).

MMSE is a brief structured screening test for global cognition. It consists of 30 items divided into 6 areas: orientation in time and space; memory (repetition of three words), attention and calculation (serial subtraction or forward/backward spelling, recall of words previously memorized); language (recognition of two objects, repetition of a short sentence; sentence comprehension; sentence writing), and constructional praxis (design copy). The score is given by the number of items completed correctly (0 to 30). Lower scores indicate worse performance and greater cognitive impairment (Folstein et al., 1975).

DSST is a Wechsler Adult Intelligence Scale-Revised (WAIS-R) subtest for attention and psychomotor speed (Wechsler, 1981). It consists of 93 small blank squares (presented in seven rows), each of which is paired at random with one of nine numbers (1 to 9) printed directly above it. A printed “key” that pairs the numbers 1 through 9 with unknown symbols is located above the row of blank squares. After a brief series of practice trials, the subject must utilize the “key” to swiftly complete the 90-s task by using the symbol that is paired with the number above each blank square, working from left to right across the rows. The measure of interest is the number of blank squares that are accurately filled in within the allotted time (maximum raw score = 93) (Wechsler, 1981).

Trail Making Test part B is a test of attentional set-shifting. Subjects are given a new sheet with a scattered set of numbers (1 to 13) and letters (A to L) circled on it and are asked to connect the numbers and letters alternately and in sequential order. The subject’s performance is judged by the time it takes to complete each path and the number of errors of commission and omission (Reitan, 1958).

Based on the results of the Cox regression model obtained by Tuena et al. (2023) regarding features with prognostic value on conversion from aMCI to AD-dementia, we considered the following three neuropsychological tests for our analyses: MMSE (Folstein et al., 1975), DSST (Wechsler, 1981),and TMT-B (Reitan, 1958).

In our study, the neuropsychological test scores mentioned above were extrapolated from the ADNI aggregated dataset (ADNIMERGE.csv) using the terms (MMSE, DIGITSCOR, TRABSCOR) and referring to ADNI1 protocols. See Table 1 for descriptive statistics of neuropsychological tests at baseline in the two populations for the final conversion class.

### 2.3 Machine learning

To explore the trends of neuropsychological functions over time according to gait profiles we used ML algorithms as in a similar study (Ghoraani et al., 2021).

The development and evaluation of the ML algorithms were conducted in a Python 3.10.9 environment, using Anaconda 23.3.1 and Jupyter Notebook 6.5.2. The following Python packages were used: numpy, pandas, matplotlib, and scikit-learn. The ML framework used was scikit-learn and optuna. ML algorithms implemented in Python were applied to identify the most accurate models in classifying aMCI subjects into two groups: aMCIs and aMCIc.

We used the following features to train the chosen algorithms: neuropsychological test scores at baseline (MMSE, DSST, and TMT-B) and the gait profile at baseline (normal/abnormal). Imputation was performed for neuropsychological test scores with missing values using KNNImputer. Categorical variables were dichotomized using one-hot encoding, with 1 indicating the occurrence of that class and 0 the occurrence of any other class of the variable. Once the features were transformed into continuous variables, they were standardized [mean = 0, standard deviation (SD) = 1] using StandardScaler.

We used 6 supervised ML techniques: Decision Tree (DT), Random Forest (RF), Gradient Boosting (GB), Support Vector Machine (SVM), Logistic Regression (LR), k-Nearest Neighbors (kNN). From the entire dataset, information from 70% of the subjects was randomly extracted and used for the training phase performed by the 6 algorithms separately. After the training phase, the three algorithms (SVM, LR, and kNN) were chosen to optimize the hyperparameters through the three main techniques (Randomized Search, Bayesian Search, and Grid Search) (Yu and Zhu, 2020). To measure the performance of the created models, 5-fold cross-validation with 5 repeats and 10-fold cross-validation with 5 repeats were performed at each step from which the best-performing models were chosen. Cross-validation was performed at 5 and 10-folds with 5 repetitions to see which of the two we would get the best metrics. After that, the best-performing algorithm was chosen and tested on the remaining of the dataset.

To evaluate the importance of the predictors for the optimized SVM algorithm in the training and testing set, we calculated the weights associated with the SVM coefficients. These weights indicate how much each feature contributes to the decision-making process of the SVM algorithm. The range of values is −1 to 1 (Bron et al., 2015). Positive weights suggest that a feature contributes to the correct classification of the specific class, while negative weights indicate a negative contribution.

The following evaluation metrics were considered for each phase of the training and test set: accuracy, precision, sensitivity (or recall), specificity, F_1_ score, and Area Under the Curve - Receiver Operating Characteristic (AUC-ROC). The Phyton ML code is available in Supplementary material 1.

## 3 Results

At baseline, the aMCIc group already showed an initial decline in cognitive measures, in particular, MMSE and DSST scores were lower and the execution time for the TMT-B was higher compared to aMCIs. In contrast, there were no significant differences in the sociodemographic variables and gait type.

### 3.1 Training

Model performance evaluation measures during training and testing were calculated for the combination of neuropsychological features with the gait profile (normal/abnormal). Initially, a baseline model (via Dummy Classifier) was trained to compare the training performance of the 6 algorithms (DT, RF, GR, SVM, LR, kNN) on 70% of the dataset. The six models were trained and evaluated through 5/10-fold cross-validation with five repeats. The SVM and LR algorithms were chosen as they are the two with the values at the highest metrics. kNN was also chosen as it has values close to SVM and LR and has the second-highest value for specificity. See Table 2 for specific evaluation metrics.

**TABLE 2:** Results of 5-fold cross-validation with 5 repeats for the six algorithms (DT, RF, GR, SVM, LR, kNN).

	DT	RF	GR	SVM	LR	kNN
Accuracy	0.53 (0.06)	0.58 (0.05)	0.57 (0.06)	0.63 (0.07)	**0.64** (0.07)	0.59 (0.07)
AUC-ROC	0.52 (0.06)	0.60 (0.07)	0.58 (0.07)	**0.65** (0.09)	**0.65** (0.08)	0.60 (0.08)
Specificity	0.44 (0.11)	0.45 (0.11)	0.46 (0.12)	0.42 (0.10)	**0.51** (0.10)	0.49 (0.13)
Sensitivity	0.59 (0.09)	0.68 (0.08)	0.65 (0.09)	**0.80** (0.12)	0.74 (0.11)	0.67 (0.11)
Precision	0.59 (0.05)	0.63 (0.05)	0.62 (0.05)	0.63 (0.04)	**0.66** (0.05)	0.63 (0.06)
F_1_ score	0.59 (0.06)	0.65 (0.05)	0.63 (0.06)	**0.70** (0.07)	0.69 (0.07)	0.64 (0.07)

DT Decision Tree, RF Random Forest, GB Gradient Boosting, SVM Support Vector Machine, LR Logistic Regression, kNN k-Nearest Neighbors. Bold values show the best results across the metrics.

**FIGURE 2:**
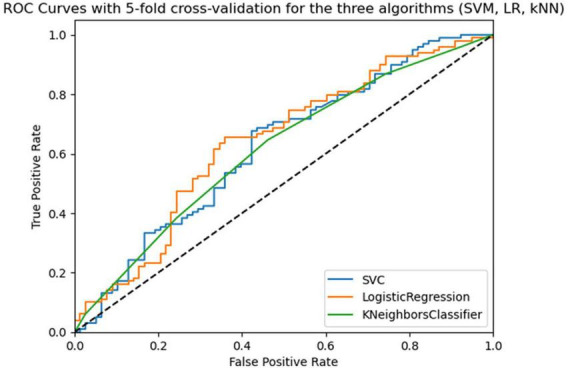
Roc curves of the three algorithms (SVM, LR, kNN) in 5-fold cross-validation with 5 repeats in the training phase.

As can be seen from Table 3 SVM and LR models were obtained at 5-fold cross-validation with 5 repeats similar to AUC-ROC (0.66 and 0.65) and similar accuracy (0.63 and 0.64), but the lowest values for all three models concern specificity. Figure 2 shows the Receiver Operating Characteristic (ROC) curves for the three algorithms (SVM, LR, and kNN). To improve the performance of the 3 models, the best hyperparameters were searched through the three main methods, for which cross-validation was performed and the optimization method that improved the algorithms with the highest metrics values (RandomizedSearchCV) was chosen. Next, the performance of the optimized algorithms was trained on 70% of the dataset and was evaluated by 5/10-fold cross-validation with 5 repetitions. As can be seen from Table 3, the performance of all three algorithms improved for the evaluation metrics with the lowest values, although specificity remained low for the LR and kNN algorithms.

**TABLE 3:** Results of 5-fold cross-validation with 5 repeats for the three algorithms chosen (SVM, LR, and kNN).

				Optimized algorithms		
	**SVM**	**LR**	**kNN**	**SVM**	**LR**	**kNN**
Accuracy	0.63 (0.07)	0.64 (0.07)	0.59 (0.07)	**0.67** (0.08)	0.64 (0.06)	0.63 (0.06)
AUC-ROC	0.65 (0.09)	0.65 (0.08)	0.60 (0.08)	**0.66** (0.04)	0.65 (0.05)	0.64 (0.04)
Specificity	0.42 (0.10)	0.51 (0.10)	0.49 (0.13)	**0.60** (0.09)	0.40 (0.07)	0.45 (0.06)
Sensitivity	0.80 (0.12)	0.74 (0.11)	0.67 (0.11)	0.72 (0.08)	**0.83** (0.11)	0.78 (0.10)
Precision	0.63 (0.04)	0.66 (0.05)	0.63 (0.06)	**0.70** (0.06)	0.64 (0.03)	0.64 (0.03)
F_1_ score	0.70 (0.07)	0.69 (0.07)	0.64 (0.07)	0.71 (0.07)	**0.72** (0.05)	0.70 (0.06)

SVM, Support Vector Machine; LR, Logistic Regression; kNN, k-Nearest Neighbors. Bold values show the best results across the metrics.

**FIGURE 3:**
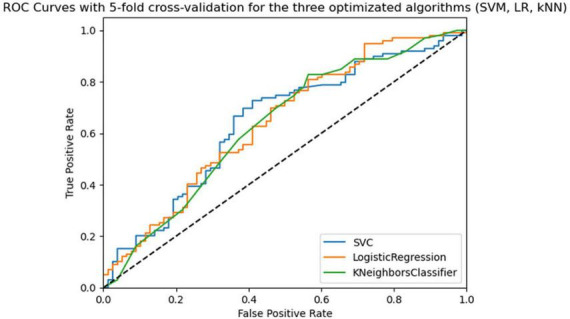
Roc curves of the three algorithms (SVM, LR, kNN) optimized in 5-fold cross-validation with 5 repeats in the training phase.

The best algorithm with the best hyperparameter optimization was SVM with an average accuracy of 0.67 (SD = 0.07), an average AUC-ROC of 0.66 (SD = 0.04), and a balance between sensitivity and specificity. Figure 3 shows the ROC curves for the three optimized algorithms. The SVM model has the highest ROC curve, indicating a higher ability to discriminate positive cases than the others.

### 3.2 Testing

**FIGURE 4:**
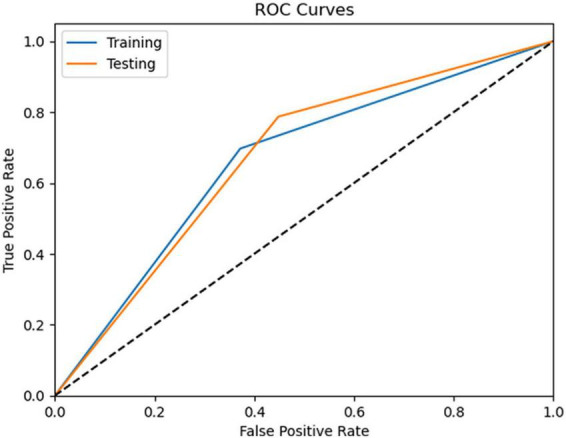
Roc curves of the SVM algorithm during training and testing.

The testing phase was performed only for the best performing model, SVM, for which we evaluated its performance using an independent set of data (30%) from the training set. The results show an accuracy of 70%, highlighting the model’s ability to correctly classify most of the cases in the test set (see Table 4). AUC-ROC is 0.67, indicating a fair result (see Figure 4). The model is able to correctly identify to make predictions about predicted positives (precision = 74%) and correctly identify true positives (sensitivity = 79%) but can identify true negatives less (specificity = 55%). Overall, the testing results show that our SVM model can provide accurate predictions, especially for cases (i.e., aMCIc).

**TABLE 4:** Results of the testing set for the optimized SVM model.

	Testing set
	**SVM**
Accuracy	**0.70**
AUC-ROC	**0.67**
Specificity	**0.55**
Sensitivity	**0.79**
Precision	**0.74**
F_1_ score	**0.76**

SVM, Support Vector Machine.

### 3.3 Predictors importance

Importance feature is a common technique to assess the importance of features for a model. However, this technique does not apply to non-parametric models such as SVM.

For the evaluation of all features, we calculated the absolute values of the coefficients of the optimized SVM algorithm to be able to evaluate the contribution of each feature in this classification. As can be seen in Table 5, MMSE was the neuropsychological test that obtained the highest weight in both training and testing. The gait type was found to have a significant weight only in the testing phase.

**TABLE 5:** Results for importance features in the training and testing set for the optimized SVM.

	Training	Testing
	**SVM**	**SVM**
MMSE	0.37	0.61
DSST	0.03	0.04
TMT-B	0.00	0.00
GAIT	0.01	0.56

SVM Support Vector Machine. Range of values: −1 and 1.

## 4 Discussion

In this paper, we sought to explore the predictive power of four predictors: gait (assessed by visual examination during neurological exam), MMSE, DSST, and TMT-B. These variables were previously identified (Tuena et al., 2023) as critical for the conversion from aMCI to AD within a follow-up period of 36 months.

To our knowledge, this is the first research that using previously published results, uses ML to study the impact of gait and gait-related cognitive measures to forecast the diagnosis of AD in aMCI. Our results show that the best algorithm was SVM and that on baseline assessment it was able to provide a 70% predictive accuracy of future AD diagnosis within 36 months from the first visit. This is true, especially for identifying cases (i.e., aMCI patients that during the 36-month follow-up converted to AD). That is to say that our model and best algorithm can be used as a quick, low-cost, and preliminary assessment for aMCI individual at high risk of developing AD, which might require more expansive and invasive testing (e.g., neuroimaging, genetic testing, cerebrospinal fluid testing). Additionally, this would enable to program in advance of secondary prevention strategies and therapies. Importance variable analysis showed that MMSE was the most important predictor during training and testing of the model, whereas gait was crucial for the testing phase of the model alone.

Many authors have used the ADNI database to identify predictors to forecast AD diagnosis using ML algorithms (Massetti et al., 2022; Rye et al., 2022; Franciotti et al., 2023; Sarica et al., 2023).

The features that have been used in the literature in this classification can be clustered into four groups: neuropsychological measures only (Battista et al., 2020; Massetti et al., 2022), neuropsychological measures and biomarkers (Chun et al., 2022; Massetti et al., 2022), neuropsychological measures and neuroimaging (Rye et al., 2022; Franciotti et al., 2023), and finally neuropsychological measures, biomarkers, and neuroimaging (Sarica et al., 2023). The study (Massetti et al., 2022) used all ADNI stages for training and compared the combination of various features to find the best performing model also stratifying it by age range, specifically for the 75–78 range and relying only on the neuropsychological assessment they obtained an accuracy of 0.72 at testing. Our accuracy score (0.70) in testing is in line with them; in particular, we demonstrate that is possible to obtain good results with a restricted set of predictors linked to AD development. Indeed, the sensitivity of our models (see Tables 2, 3) is higher than the one reported (0.5) by Masetti and co-authors for neuropsychology tests alone (Massetti et al., 2022). It is important to note that multidimensional features datasets yield higher accuracies (e.g., 0.87; Sarica et al., 2023) and should be preferred. Yet, a meta-analysis (Battista et al., 2020) that evaluated ML approaches used in AD illustrated the ranges of performance metrics of studies that predicted conversion from MCI to AD based on neuropsychological tests: accuracy = 0.61–0.85, sensitivity = 0.50–0.91, specificity = 0.48–0.91, AUC-ROC = 0.67–0.93. Moreover, the work by Battista et al. (2020) found that the best tests to predict the conversion to AD in MCI included MMSE and TMT-B in addition to memory tests. However, as found in a study (Tuena et al., 2023) memory tests are not affected by gait, and we preferred to focus on the cognitive measures linked to gait. Our results show that it is possible to have comparable accuracy, sensitivity, and specificity using a restricted set of neuropsychological gait-related predictors that are linked to the condition of interest. Nevertheless, a great challenge of ML research is to improve specificity parameter for aMCIc and aMCIs. This was noted in our results and also in the meta-analytic work by Battista and co-authors (Battista et al., 2020).

Feature weights in an SVM model indicate the relative importance of distinctive features in ML model decision making. Although these weights are not statistically significant values in the traditional sense, nevertheless they are valuable tools for model interpretation. For example, in neuroimaging studies, features are often considered as brain voxels to identify the brain regions that contribute most to the diagnosis or course of a disease (e.g., AD-dementia) and show them graphically by weight-map (Bron et al., 2015). Our analysis of the SVM model weights shows that MMSE was the predictor that contributed most positively to our classification problem and that gait contributed only to the testing phase. Because the test set represents a different portion of the data (30%) than the training set, it is possible that features take on different importance in the testing phase. This suggests that gait type might play an important role in the conversion phase on a different sample than the one used for training the model. In line with the previous study (Tuena et al., 2023), we found that the most important predictor was MMSE in the training and testing phase and that the type of gait, within this feature cluster, contributes to the prediction of conversion. One longitudinal study (Chun et al., 2022) used ML to forecast dementia diagnosis in aMCI using sociodemographic, neuropsychological, and APOE variables as features. Among the features they found important for their best algorithm was MMSE. We showed that gait could be a critical predictor to improve ML performance for future AD diagnosis. This finding is also in line with embodiment theories in aging, which suggest that sensorimotor and perceptual impairments could hamper higher-order cognitive functions and be linked to neurodegenerative processes (Kuehn et al., 2018).

ML techniques can be a powerful method to overcome the limits of classic statistical approaches (Tuena et al., 2020). Indeed, despite p-value being a widely used and consolidated parameter, it lacks predictive information; crucially, ML methods enable to generalize findings of diagnostic, prognostic, and predictive studies to new observations and inform theory and practice (Dwyer et al., 2018). Therefore, it is suggested to validate research studies with both classical and ML statistical approaches. Following this line of reasoning, we provided scientific evidence that previously identified predictors of AD diagnosis can have promising predictive diagnostic value.

However, our study has some limitations that we must acknowledge. First, the results can be only generalized to aMCI patients with inclusion/exclusion criteria of the ADNI1 cohort. Second, the performance of the classification accuracy is still not high enough, nevertheless considering that we used only four quick-to-administer predictors to forecast AD diagnosis within 36 months from the first visit, our results are encouraging. Third, regarding the classification of gait type is numerically unbalanced, within aMCI, and this may have negatively affected the evaluation of permutation importance. Fourth, the categorization of the gait type from the ADNI database is dichotomous categorical. Future research could improve these results by adopting quantitative (e.g., accelerometer) gait measures to provide ML with numeric patterns. Then, the model could be tested on other public and private MCI databases to assess its generalizability. Lastly, more complex algorithms such as neural networks and deep learning algorithms could be tested to improve accuracy performance.

To conclude, in this ML study we provided additional evidence that previously identified predictors with classic statistical approaches can have promising and encouraging results using artificial intelligence. Moreover, we showed that in addition to established neuropsychological tests, gait, and in general the motor system, could contribute to cognitive deterioration and AD diagnosis, endorsing theories that suggest a link between mind and body.

## Data availability statement

The original contributions presented in this study are included in this article/Supplementary material, further inquiries can be directed to the corresponding author.

## Ethics statement

The studies involving human participants were reviewed and approved by the institutional review boards of the participating institutions in the Alzheimer’s Disease Neuroimaging Initiative (ADNI). This study will be conducted in accordance with good clinical practice guidelines, the Declaration of Helsinki, US 21CFR Part 50–Protection of Human Subjects and Part 56–Institutional Review Boards, and in compliance with state and federal HIPAA regulations. Written informed consent was obtained from all subjects and/or authorized representatives and study partners before protocol-specific procedures were carried out. The Ethics committees/institutional review boards that approved the ADNI study are: Albany Medical Center Committee on Research Involving Human Subjects Institutional Review Board, Boston University Medical Campus and Boston Medical Center Institutional Review Board, Butler Hospital Institutional Review Board, Cleveland Clinic Institutional Review Board, Columbia University Medical Center Institutional Review Board, Duke University Health System Institutional Review Board, Emory Institutional Review Board, Georgetown University Institutional Review Board, Health Sciences Institutional Review Board, Houston Methodist Institutional Review Board, Howard University Office of Regulatory Research Compliance, Icahn School of Medicine at Mount Sinai Program for the Protection of Human Subjects, Indiana University Institutional Review Board, Institutional Review Board of Baylor College of Medicine, Jewish General Hospital Research Ethics Board, Johns Hopkins Medicine Institutional Review Board, Lifespan–Rhode Island Hospital Institutional Review Board, Mayo Clinic Institutional Review Board, Mount Sinai Medical Center Institutional Review Board, Nathan Kline Institute for Psychiatric Research & Rockland Psychiatric Center Institutional Review Board, New York University Langone Medical Center School of Medicine Institutional Review Board, Northwestern University Institutional Review Board, Oregon Health and Science University Institutional Review Board, Partners Human Research Committee Research Ethics, Board Sunnybrook Health Sciences Centre, Roper St. Francis Healthcare Institutional Review Board, Rush University Medical Center Institutional Review Board, St. Joseph’s Phoenix Institutional Review Board, Stanford Institutional Review Board, The Ohio State University Institutional Review Board, University Hospitals Cleveland Medical Center Institutional Review. Board, University of Alabama Office of the IRB, University of British Columbia Research Ethics Board, University of California Davis Institutional Review Board Administration, University of California Los Angeles Office of the Human Research Protection Program, University of California San Diego Human Research Protections Program, University of California San Francisco Human Research Protection Program, University of Iowa Institutional Review Board, University of Kansas Medical Center Human Subjects Committee, University of Kentucky Medical Institutional Review Board, University of Michigan Medical School Institutional Review Board, University of Pennsylvania Institutional Review Board, University of Pittsburgh Institutional Review Board, University of Rochester Research Subjects Review Board, University of South Florida Institutional Review Board, University of Southern, California Institutional Review Board, UT Southwestern Institution Review Board, VA Long Beach Healthcare System Institutional Review Board, Vanderbilt University Medical Center Institutional Review Board, Wake Forest School of Medicine Institutional Review Board, Washington University School of Medicine Institutional Review Board, Western Institutional Review Board, Western University Health Sciences Research Ethics Board, and Yale University Institutional Review Board.

## Author contributions

CT: Conceptualization, Formal analysis, Methodology, Writing –review and editing. CP: Formal analysis, Writing –original draft, Writing –review and editing. CS-B: Writing –original draft. MS-B: Supervision, Writing –review and editing. GR: Supervision, Writing –review and editing.

## Footnotes

1
http://adni.loni.usc.edu/

2
http://www.adni-info.org/

3
https://www.adni3.org/locations

4
https://adni.loni.usc.edu/methods/documents/
